# What Is the Most Accurate Method for the Treatment of Diminutive Colonic Polyps? Standard Versus Jumbo Forceps Polypectomy

**DOI:** 10.1097/MD.0000000000000621

**Published:** 2015-04-17

**Authors:** Fatih Aslan, Cem Cekiç, Mehmet Camci, Emrah Alper, Nese Ekinci, Zehra Akpinar, Serkan Alpek, Mahmut Arabul, Belkis Unsal

**Affiliations:** From the Department of Gastroenterology, KatipCelebi University Ataturk Training and Research Hospital, Izmir, Turkey.

## Abstract

Different methods such as standard, hot, and jumbo forceps are used in endoscopic treatment of diminutive colon polyps. In the current study, it was aimed to compare efficacy and safety of standard and jumbo forceps polypectomy methods in treatment of diminutive colon polyps of ≤5 mm. Polyps with ≤5 mm which were excised during colonoscopy by using standard or jumbo forceps were evaluated. Standard and jumbo forceps polypectomy methods were randomly performed in 212 consecutive patients with diminutive colorectal polyp. One-bite polypectomy and complete resection rates were also determined among polypectomy methods. Results of 161 standard forceps polypectomy and 102 jumbo forceps polypectomy were retrospectively evaluated. Both one-bite polypectomy and complete resection rates were significantly higher in the jumbo forceps polypectomy group than the standard forceps polypectomy group (*P* < 0.001). In the subgroup analysis performed according to polyp sizes, complete resection rate among polyps with 3-mm diameter was determined as 100%. However, numbers of bites in 4-mm and 5-mm polyps were higher in the standard forceps polypectomy group, and complete resection rate was lower than in the jumbo forceps polypectomy group (*P* < 0.001). Both endoscopic treatment methods may be employed in treatment of diminutive colon polyps with ≤5 mm. However, jumbo forceps polypectomy is a more effective treatment method in 4- to 5-mm polyps with high one-bite polypectomy and complete resection rate.

## INTRODUCTION

The main objective of colonoscopy, which is used to decrease colon cancer risk, and as a golden standard screening method, is to determine premalign or malign lesions, and to treat endoscopically, if there are any.^1,2^ The majority of polyps having malignancy potential for colon cancer can be removed endoscopically.^2^ According to their sizes and their endoscopic appearances, polyps may be removed by one of endoscopic treatment methods such as cold and hot forceps polypectomy, cold or hot snare polypectomy, endoscopic mucosal resection (EMR), and endoscopic submucosal dissection.^3^

Different methods such as standard, hot, and jumbo forceps polypectomy (JFP) may be used in treatment of colon polyp ≤5 mm, named as diminutive polyps.^4^ If a diminutive polyp is encountered during colonoscopy, selection of endoscopic method depends on experience and preference of the endoscopist. In a study performed among American gastroenterologists, 50.3% of participants preferred forceps polypectomy method in polyps with 1- to 3-mm size, whereas 18.5% preferred cold forceps polypectomy in polyps with 4- to 6-mm size.^5^ Although it is reported in recent years that Jumbo forceps, which is larger in size than standard forceps, is an effective treatment modality in small size colon polyps, there is still no consensus in endoscopic treatment of diminutive polyps.^6^ Moreover, there is no study comparing efficacies of SFP and JFP methods in patients with 3- to 5-mm polyp sizes.

Our aim in the current study was to investigate efficacy and safety of SFP and JFP methods in diminutive colon polyps.

## METHODS

### Ethical Considerations

This study was approved by the local ethics committee of KatipCelebi University. Written informed consents were obtained from all patients before the procedure. The study concept, hypothesis, and design were investigator initiated, and no financial support or free devices were received.

### Patients and Patients’ Selection

Consecutive patients who had colonoscopy at the Department of Gastroenterology, Ataturk Training and Research Hospital, KatipCelebi University, Turkey, between dates April 2012 and December 2013, were eligible for the study.

Patients older than 40 years of age who were scheduled to undergo screening or surveillance colonoscopy and who had at least 1 eligible polyp were included in the study. An eligible polyp was defined as a polyp measuring 3 to 5 mm in size. Patients with inflammatory bowel disease, polyposis syndromes, and taking antiaggregate and anticoagulant drugs were excluded from the study.

### Study Design

Patient data were prospectively recorded, and patients who had diminutive polyps with 3- to 5-mm diameter and had their polyps removed were retrospectively evaluated. Polyps were divided into 3 groups according to their sizes: 3, 4, and 5 mm in diameter. Analyses were performed on one-bite and complete resection rates during SFP and JFP procedures, pathological evaluation, and colonoscopy records. Also preparation time for colonoscopy procedures, colonoscopy preparation quality, number and location of polyps, procedure duration, and complication frequency were compared between different endoscopic polypectomy procedures. Two hundred and twelve consecutive patients with small colorectal polyps, 3 to 5 mm in size were randomized into the SFP and JFP groups.

### Procedure

**FIGURE 1:**
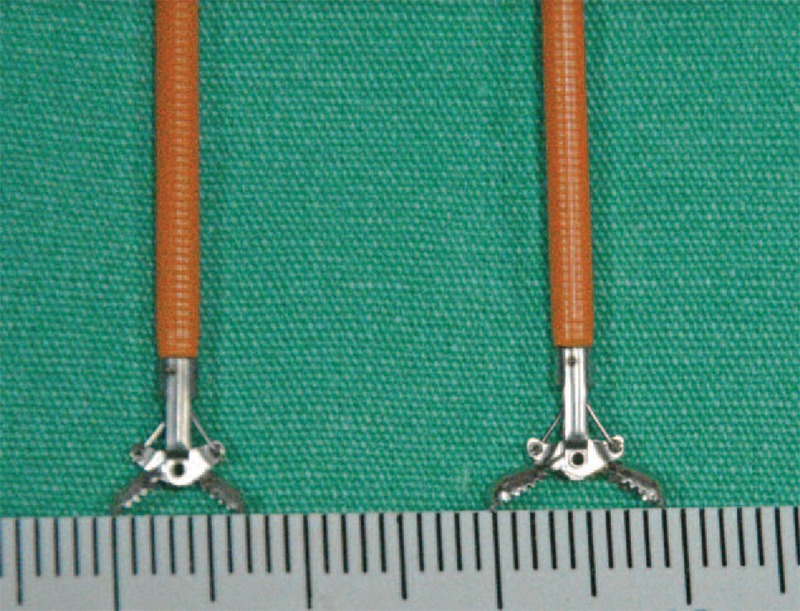
Standard biopsy forceps and jumbo biopsy forceps image.

The same gastroenterologist alone performed all of procedures. The procedure was a polypectomy with either a standard biopsy forceps (M00513402, Radial Jaw 4-Standard capacity forceps, Boston Scientific, MA) or a jumbo biopsy forceps (M00513362, Radial Jaw 4-jumbo forceps, Boston Scientific, MA) (Figure 1), and all procedures were performed with the Olympus-H180 AL (Olympus, Tokyo, Japan) or Pentax EC-3890 LK (Hoya, Tokyo, Japan).

The patients were sedated with intravenous midazolam (0.05 mg/kg) and propofol (0.5 mg/kg) just before the procedure, and the oxygen saturation and electrocardiograms were monitored. The sedation procedure was performed by an experienced anesthesiologist.

The sizes of the polyps were measured by visual comparison with the open standard or jumbo forceps. Then, the base of polyp was bitten and removed so that the polyp was removed as a whole by standard or jumbo forceps. Blood was washed by water-jet washing of the polypectomy area, which was also controlled by conventional and narrow band imaging (NBI) or I-scan. If the polyp was removed by performing one bite, then it was recorded in the electronic form as “one-bite polypectomy.” If residual tissue was left, the remaining polyp parts were bitten again until the remnants were removed completely by the same forceps. After each bite, polypectomy area was controlled by water-jet washing. After polyp removal, number of bites and excised tissue samples were recorded in the electronic form.

Resected polyps were placed in a formalin container for histopathological examination so as to be evaluated by experienced pathologists. Complete resection decision was given as the result of histopathological evaluation.

### Statistical Analysis

We used the χ^2^ or Fisher exact test to compare the success rates between the groups. To compare continuous or discrete variables between 2 groups, we used a 2-sample *t* test or Mann-Whitney *U* test. The level of significance was accepted as *P* < 0.05. Data were analyzed by using SPSS 17.0 program (SPSS Inc, Chicago, IL).

## RESULTS

A total result of 263 polypectomy procedures (SFP group = 131 and JFP group = 81 patients) were analyzed. The patients’ demographic characteristics, bowel preparation quality, cecum intubation rate, and intubation rate of the terminal ileum were similar between both groups (Table 1). None of the patients dropped out from the study.

**TABLE 1:**
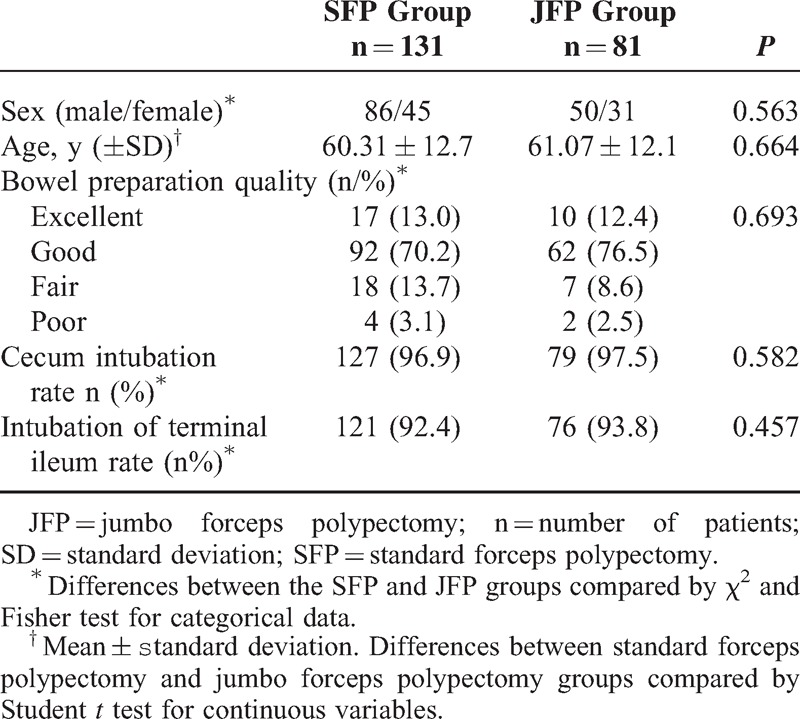
Baseline Characteristics, Indications, and Outcomes in Patients With standard Forceps Polypectomy and Jumbo Forceps Polypectomy

**FIGURE 2:**
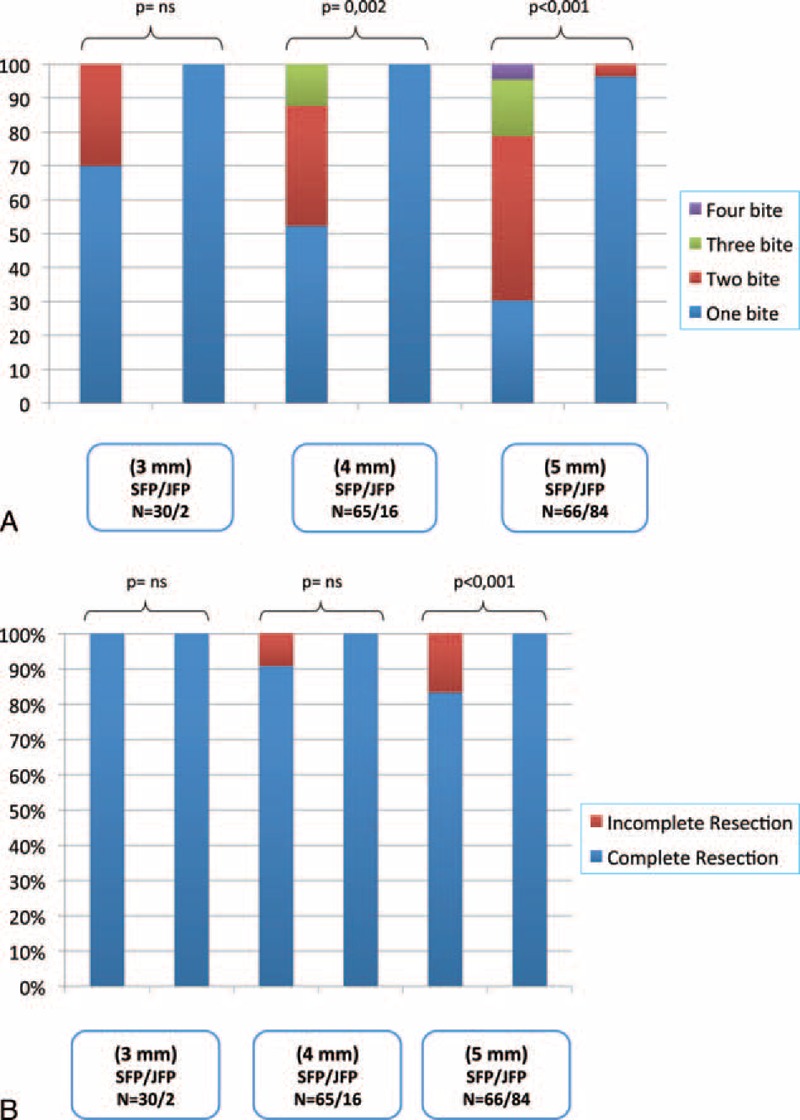
(A) Number of bites during removal of polyps with sizes 3, 4, and 5 mm by the standard forceps polypectomy or jumbo forceps polypectomy method. (B) Complete and incomplete resection rates of the standard forceps polypectomy and jumbo forceps polypectomy methods performed in treatment of polyps with 3, 4, and 5 mm in diameter. JFP = jumbo forceps polypectomy; SFP = standard forceps polypectomy.

Numbers of performed polypectomy were 161 and 102 in the SFP and JFP groups, respectively. No significant difference was defined between the SFP and JFP groups in polyp locations (*P* = 0.168) and postoperative bleeding rates (*P* = 0.496). No perforation due to endoscopic procedure or polypectomy was seen. Although the frequency of adenomatous polyps in the JFP group was higher than the SFP group no significant difference was found statistically (*P* = 0.077). In the JFP group, polyps were larger in size and the dysplasia stage was higher than in the SFP group (*P* < 0.001 and *P* = 0.013, respectively). Both one-bite and complete resection rates were higher in the JFP group than the SFP group (*P* < 0.001). The number of bites to achieve endoscopic complete resection was higher in the SFP group (Table 2, Figure 2A, B).

**TABLE 2:**
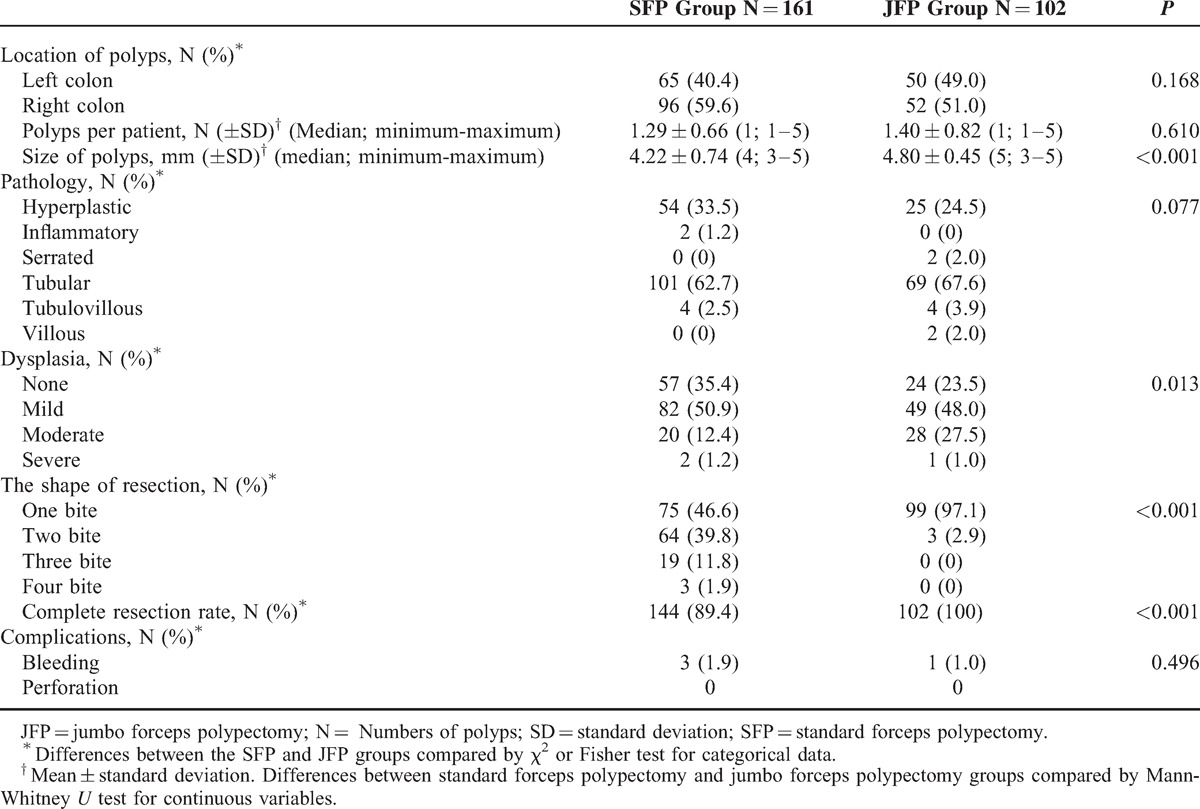
Comparison of Polyps in Patients With Standard Forceps Polypectomy and Jumbo Forceps Polypectomy

When intergroup analysis was performed between the groups in polyp sizes, one-bite polypectomy rate in the SFP group in 3-, 4-, and 5-mm polyps were 70% (N = 21), 52.3% (N = 34), and 30.3% (N = 20) in the same order, and in the JFP group were 100% (N = 2), 100% (N = 16), and 96.4% (N = 81) (*P* > 0.05, *P* = 0.002, and *P* < 0.001 in the same order) (Figure 2A, B).

If complete resection rates were evaluated according to polyp sizes, complete resection rate in 3-mm polyps was 100% in both the SFP and JFP groups. However, the resection rates were lower in the SFP group with 4- and 5-mm polyps when compared with those in the SFP group (90.8%, 83.3% versus 100%, 100%; *P* = 0.255 and *P* < 0.001) (Figure 2A, B).

## DISCUSSION

Data were prospectively recorded in our retrospective trial, and efficacy and safety of SFP and JFP were investigated in 263 polypectomy procedures.

Colonoscopy and polypectomy procedures which help in early diagnosis and treatment of colorectal polyps are important minimal invasive procedures decreasing colorectal cancer risk.^1^ Diminutive colon polyps which have malignancy potential are commonly encountered during colonoscopy.^7^ It has been reported that there is advanced adenoma at 6.75% to 8.7% rate in diminutive and small colon polyps.^8,9^ These results indicate that effective and safe treatment methods are required in the treatment of small polyps. There are various polypectomy methods such as standard, hot, and jumbo biopsy, cold snare, and hot snare. Polypectomy methods preferred by endoscopists vary generally according to the polyp size. In a questionnaire study which investigated the preferred polypectomy methods among 189 endoscopists, it was reported that polypectomy methods differed according to the polyp sizes. In the same study, it was determined that cold forceps polypectomy technique was preferred in treatment of polyps with 1-, 3-, 3- to 6-, and 6- to 9-mm diameters in the decreasing rate.^5^ These techniques had lower complication rates when compared with rapid and easily applicable hot snare and hot biopsy methods.^6,9^ Polypectomy methods used in 1- to 6-mm polyps vary extremely among endoscopists.^5^ One reason of different preferences might be due to different complete resection rates among diminutive polyps. In a study investigating efficacy of SFP in treatment of diminutive colon polyps, it was reported after evaluation of polypectomy area following polyp removal by EMR that complete resection was performed at 39% rate.^10^ Jung et al reported in their study that complete resection rates were 92.3% in all diminutive polyps, and 100% in polyps with 1- to 3-mm diameter.^11^ Results of prospective studies were different in efficacy of the SFP method. In the current study, complete resection rates were 89.4% and 100% in the SFP and JFP groups, respectively (*P* < 0.001). When subgroup analysis was performed in the SFP group, complete resection rates were 100%, 90.8%, and 83.3% in polyps with 3, 4, and 5 mm in diameter reciprocally.

There are some disadvantages of SFP technique such as low one-bite polypectomy and complete resection rates, and increased risk of residual tissue.^4,10,12^ One of the reasons might be inadequate examination of polypectomy area because of bleeding after the first bite.^4^ In our study, complete resection rate was high in the SFP group when compared with the literature. However, it indicated that more bites were required to obtain this resection rate (Figure 2A). The high complete resection rate in our SFP group might be interpreted as biting was continued until polyps were completely resected; washing of the procedure area was repeated after each biting; and NBI examination was performed.

Due to these disadvantages in SFP technique, biopsy forceps with wider mouth may be preferred to remove polyps as a whole without any residual tissues as well as to obtain complete resection.^13^ Draganov et al performed a study comparing large capacity forceps and jumbo forceps in treatment of small colon polyps with ≤6-mm diameter. They showed prospectively that one-bite polypectomy and complete resection rates were higher in the JFP group.^6^ In the current study, one-bite polypectomy and complete resection rates were 100% and 96.4% in polyps with 3- and 4-mm diameters in the JFP group.

Which method should be preferred according to polyp size? The unique point in our study is that this is the first study comparing efficacies of 2 methods in polyps with 3- to 5-mm diameter. Similar to the literature we determined that one-bite polypectomy and complete resection rates in the JFP group were significantly higher in our study. Different from the literature, we showed that one-bite polypectomy rate was low in 3-mm polyps if SFP was preferred, and incomplete resection might be performed at 9.2% rate although more bites were performed in treatment of polyps with 4-mm diameter (Figure 2A, B).

Performing more bites until being sure that polyp is completely removed may increase bleeding risk. It was reported in a prospective study investigating SFP methods that bleeding was observed in 6 patients (4.29%).^6^ Additionally, it was reported that anticoagulant drug use history caused increased bleeding risk after cold polypectomy methods.^8^ In the current study, although the number of patients in the SFP group with bleeding complication was higher, there was no statistical significance between the groups. The low bleeding rate in our study may be explained by absence of anticoagulant drug use history in the cohort; small size of polyps; and water-jet washing performed during the procedure.

One of the preferred treatment methods in small colon polyps is hot forceps polypectomy technique.^14^ The preference rates for hot forceps polypectomy also differ among endoscopists.^5^ It was reported that delayed bleeding, and postpolypectomy syndrome secondary to electrocoagulation might be encountered by using hot biopsy forceps. It was reported that hot biopsy forceps technique might cause delayed perforation in especially ascending colon and cecum, because their walls were very thin.^15^ Moreover, cautery artifact observed after hot forceps polypectomy procedure caused difficulty in histopathological evaluation.^16^ Peluso et al reported that 17% of residual tissue was remained in colonoscopic examination performed hot biopsy polypectomy.^17^ These risks are rarely encountered in cold methods such as SFP, JFP, and cold snare polypectomy. According to our study results, SFP and JFP are reliable methods in treatment of polyps with 3- to 5-mm diameter, and no complication has been observed.

Another treatment option in treatment of small polyps is cold snare polypectomy method. Efficacy and safety of this method have been shown in large volume studies.^18,19^ In our study comparing cold and hot snare polypectomy methods, it was determined that procedure duration was shortened, and there was no unfavorable effects, such as cautery artifacts, in the cold snare method.^9^ However, performing polypectomy may be difficult if polyps are located in discrete localizations for snare method, such as in cecum, ascending colon, or behind the folds.^20^ Additionally, polyps may not be completely removed by cold snare polypectomy method and after polypectomy the polyp may be lost before it can be aspirated from the colon into the trap.^8,18,21^ In our study, polypectomy materials were examined completely by both methods. These methods seem to be performed easily and rapidly, although there is stool in every colonic location. According to the results of our study, the JFP method can be considered an easy and effective endoscopic treatment option for both removing the polypectomy material out in the forceps without losing in the colon and achieving complete resection as one piece, especially for polyps 4 to 5 mm in diameter.

There are also some limitations in our study. Firstly, this current study was a retrospective single center study. Secondly, recurrence rate was not investigated. And lastly, numbers of polyps with 3-mm diameter were not homogenously distributed between the groups. However, one-bite polypectomy and complete resection rates were high in the SFP method in diminutive polyps with 3-mm diameter, whereas in the JFP method with 4-mm diameter. Therefore, efficacy of JFP may be expected higher in polyps with 3-mm diameter.

The different aspects of our study from other studies are given as follows: procedures were performed by an experienced endoscopist without help of any fellow or trainer, and data were recoded immediately after the procedure. The other point is that this current study is the first in the literature comparing the SFP and JFP methods.

In conclusion, SFP and JFP are easy and rapidly applicable practical polypectomy methods with low complication rates. The SFP and JFP methods are effective and safe methods with high one-bite polypectomy and complete resection rates in treatment of polyps with 3-mm diameter, whereas the same is true for the JFP method in 4- and 5-mm polyps.
